# Hot Milk as a Stimulant

**Published:** 1893-06

**Authors:** 


### HOT MILK AS A STIMULANT.

No one who, fatigued by over exertion of body and mind, has ever experienced the reviving influence of a tumbler of this beverage, heated as hot as it can be sipped, will willingly forego a resort to it because of its being rendered somewhat less acceptable to the palate. The promptness with which its cordial influence is felt is indeed surprising. Some portion of it seems to be digested and appropriated almost immediately, and many who now fancy they need alcoholic stimulants when exhausted by fatigue, will find in this simple draught an equivalent that will be abundantly satisfying, and far more enduring in its effects.

